# Identifying and removing haplotypic duplication in primary genome assemblies

**DOI:** 10.1093/bioinformatics/btaa025

**Published:** 2020-01-23

**Authors:** Dengfeng Guan, Shane A McCarthy, Jonathan Wood, Kerstin Howe, Yadong Wang, Richard Durbin

**Affiliations:** b1 Department of Computer Science and Technology, Center for Bioinformatics, Harbin Institute of Technology, Harbin 150001, China; b2 Department of Genetics, University of Cambridge, Cambridge CB2 3EH, UK; b3 Wellcome Sanger Institute, Wellcome Genome Campus, Cambridge CB10 1SA, UK

## Abstract

**Motivation:**

Rapid development in long-read sequencing and scaffolding technologies is accelerating the production of reference-quality assemblies for large eukaryotic genomes. However, haplotype divergence in regions of high heterozygosity often results in assemblers creating two copies rather than one copy of a region, leading to breaks in contiguity and compromising downstream steps such as gene annotation. Several tools have been developed to resolve this problem. However, they either focus only on removing contained duplicate regions, also known as haplotigs, or fail to use all the relevant information and hence make errors.

**Results:**

Here we present a novel tool, purge_dups, that uses sequence similarity and read depth to automatically identify and remove both haplotigs and heterozygous overlaps. In comparison with current tools, we demonstrate that purge_dups can reduce heterozygous duplication and increase assembly continuity while maintaining completeness of the primary assembly. Moreover, purge_dups is fully automatic and can easily be integrated into assembly pipelines.

**Availability and implementation:**

The source code is written in C and is available at https://github.com/dfguan/purge_dups.

**Supplementary information:**

Supplementary data are available at *Bioinformatics* online.

## 1 Introduction

The superior and increasing throughput of long-read sequencing technologies, such as from Pacific Biosciences (Pacbio) and Oxford Nanopore Technologies (ONT), is revolutionizing the sequencing of genomes for new species (Phillippy, 2017). Long-read assemblers, such as Falcon (Chin *et al.*, 2016) and Canu (Koren *et al.*, 2017), typically generate haplotype-fused paths of a diploid genome, with Falcon-unzip (Chin *et al.*, 2016) further able to separate the initial assembly into primary contigs and haplotigs. However, when there is high heterozygosity as in many outbred species, for example, most insects and marine animals, the allelic relationships between haplotypic regions can be hard to identify, causing not only haplotigs to be mislabeled as primary contigs, but also overlaps to be kept among the primary contigs. The majority of these retained overlaps are between homologous chromosomes, and the resulting duplication harms downstream processes, such as scaffolding and gene annotation, leading to incorrect results.

Tools such as purge_haplotigs (Roach *et al.*, 2018) and HaploMerger2 (Huang *et al*., 2017) have been designed to resolve this problem. Purge_haplotigs makes use of both read depth and sequence similarity to identify haplotigs. However, it does not identify heterozygous overlaps, and requires users to specify read-depth cutoffs manually. HaploMerger2 seeks to identify both haplotigs and overlaps, but it ignores read depth and relies only on the alignment of contigs to each other.

Here we describe a novel purging tool, purge_dups, to resolve the haplotigs and overlaps in a primary assembly, using both sequence similarity and read depth. Purge_dups is now being used routinely in the Vertebrate Genomes Project assembly pipeline.

## 2 Materials and methods

Given a primary assembly and long-read sequencing data, we apply the following steps to identify haplotigs and overlaps. A more detailed description of the methods is available in the Supplementary Material.

1. We use minimap2 (Li, 2016) to map long-read sequencing data onto the assembly and collect read depth at each base position in the assembly. The software then uses the read-depth histogram to select a cutoff to separate haploid from diploid coverage depths, allowing for scenarios where the total assembly is dominated by either haploid or diploid sequence.
2. We segment the input draft assembly into contigs by cutting at blocks of ‘N’s, and use minimap2 to generate an all by all self-alignment.
3. We next recognize and remove haplotigs in essentially the same way as purge_haplotigs, and remove all matches associated with haplotigs from the self-alignment set.
4. Finally we chain consistent matches in the remainder to find overlaps, then calculate the average coverage of the matching intervals for each overlap, and mark an unambiguous overlap as heterozygous when the average coverage on both contigs is less than the read-depth cutoff found in step 1, removing the sequence corresponding to the matching interval in the shorter contig.

## 3 Results and discussion

We evaluated the performance of purge_dups (v1.0.0) on four Falcon-unzip primary assemblies: *Arabidopsis thaliana* (At) (Chin *et al.*, 2016), *Anopheles coluzzi* (Ac) (Kingan *et al.*, 2019),grape *Vitis vinifera* L. cv. Cabernet Sauvignon (Vv) and pinecone soldierfish *Myripristis murdjan* (Mm), and compared our results to those of purge_haplotigs (v1.0.4), HaploMerger2. The expected genome sizes and heterozygosities of these genomes calculated by GenomeScope (Vurture *et al.*, 2017) are given in Supplementary Table S1, with heterozygosity ranging from 0.6% (Ac) to 1.6% (Vv).

**Fig. 1.**
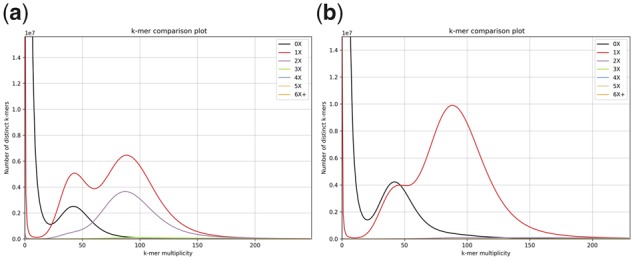
K-mer comparison plots for draft and purge_dups Mm assemblies (*k* = 21). The horizontal axis represents the copy number of k-mers in short reads from the same sample, the vertical axis shows the number of distinct k-mers and the colored lines denote k-mers which occur in the given number of times in the assembly. (**a**) The purple line shows 209.1 million two-copy k-mers accumulating in the haploid and diploid areas, which correspond to duplicated haplotigs or overlaps in the primary assembly. (**b**) Only 7.6 million two-copy k-mers remain after purging with purge_dups

K-mer comparison analysis (Mapleson *et al.*, 2017) shows that purge_dups removes 96.4% of duplicated haploid-unique k-mers in the Falcon-unzip assembly of Mm (Fig. 1). Comparable figures for HaploMerger2 and purge_haplotigs are 95.7% and 81.2% respectively (Supplementary Fig. S1) and for At are 88.4%, 87.3% and 80.7% respectively (Supplementary Fig. S2). Supplementary Figures S3 and S4 show examples of regions where purge_dups removes both contained and overlapping duplication, whereas purge_haplotigs only removes fully contained duplication.

Table 1 presents statistics on assembly and for the four assemblies, using Benchmarking Universal Single-Copy Orthologs (BUSCOs) (Simão *et al.*, 2015) to assess the consequences of purging for gene set completeness and duplication. Results are given for the original assemblies, purge_haplotigs, purge_dups and HaploMerger2 (with and without repeat masking). All purging methods remove a substantial amount of sequence from the primary assembly and decrease BUSCO duplication. No single method performs uniformly best across all assemblies and all metrics. However purge_haplotigs consistently leaves more duplicated sequence and genes. For all assemblies other than Mm, purge_dups gives the highest fraction of single-copy complete genes, and the lowest fraction of missing genes. Although purge_dups has only a limited ability to explicitly handle repeats it does not exhibit signs of significant overpurging.

**Table 1.** BUSCO scores and assembly metrics

	BUSCO scores (%)					Assembly size (Mb)	Num. Contigs
	C	C(S)	C(D)	F	M		
At-orig	**98.1**	91.9	6.2	**0.3**	**1.6**	140	172
At-PH	97.7	96.0	1.7	0.6	1.7	123	109
At-PD	97.8	**96.7**	**1.1**	0.6	**1.6**	**121**	**96**
At-HM	96.8	95.6	1.2	0.6	2.6	122	117
At-HMm	96.8	95.7	**1.1**	0.6	2.6	**121**	102

Ac-orig	98.7	94.7	4.0	0.6	0.7	266	372
Ac-PH	98.8	96.9	1.9	**0.5**	0.7	253	224
Ac-PD	**98.9**	**98.6**	0.3	0.6	**0.5**	246	**192**
Ac-HM	98.5	98.2	0.3	0.6	0.9	**245**	223
Ac-HMm	98.6	98.4	**0.2**	0.6	0.8	246	212

Vv-orig	**92.2**	79.8	12.4	**1.5**	6.3	591	718
Vv-PH	92.1	88.1	4.0	1.6	6.3	457	**259**
Vv-PD	91.9	**89.9**	2.0	1.9	**6.2**	**452**	324
Vv-HM	NA	NA	NA	NA	NA	NA	NA
Vv-HMm	91.8	**89.9**	**1.9**	1.8	6.4	458	383

Mm-orig	**95.8**	79.0	16.8	**2.0**	**2.2**	1250	1290
Mm-PH	94.5	89.1	5.4	2.4	3.1	888	517
Mm-PD	94.4	90.9	3.5	2.7	2.9	**838**	563
Mm-HM	94.6	91.3	3.3	2.5	2.9	850	600
Mm-HMm	94.7	**91.6**	**3.1**	2.6	2.7	845	**443**

Mm-origS	**95.3**	70.7	24.6	**2.2**	**2.5**	1252	764
Mm-PHS	94.7	87.5	7.2	2.5	2.8	891	**221**
Mm-PDS	94.8	91.2	3.6	2.7	**2.5**	**840**	222
Mm-HMS	94.9	91.3	3.6	2.5	2.6	852	343
Mm-HMmS	94.8	**91.6**	**3.2**	2.5	2.7	848	365

C, complete genes; C(S), complete single-copy genes; C(D), complete duplicate genes; F, fragmented genes; M, missing genes; orig, Falcon-unzip; PH, purge_haplotigs; PD, purge_dups; HM, HaploMerger2; HMm, HaploMerger2 with masking; PHS, PDS, HMS, HMmS: purge_haplotigs (respectively purge_dups, HaploMerger2 with and without repeat masking) after scaffolding and polishing. Values in bold indicate the best score of each type in each section. The HaploMerger2 run without masking on Vv did not complete.

For Mm, we also had 10X Genomics linked read data, and used this for scaffolding using Scaff10x (https://github.com/wtsi-hpag/Scaff10X). Following this with a round of polishing with Arrow closed a number of gaps, reducing contig number further and increasing contig N50. For the purge_haplotigs assembly, this resulted in 221 scaffolds with N50 8.17 Mb, and the final contig N50 3.48 Mb, whereas scaffolding the purge_dups assembly generated 222 scaffolds with N50 23.68 Mb, and contig N50 increased substantially from 2.63 Mb to 11.98 Mb. The nominal contiguity was even greater for the scaffolded HaploMerger2 masked assembly with scaffold N50 34.53 Mb, and contig N50 16.39 Mb. However, when we further assessed the scaffolds with QUAST (Gurevich *et al.*, 2013), the purge_dups scaffolds had the highest NGA50 (characteristic length of material correctly aligned to the genome) of 16.73 Mb, while HaploMerger2 scaffolds only had 7.86 Mb NGA50, with 126 scaffold misassemblies compared to 22 for purge_dups (Supplementary Table S2).

The improvements that purging makes to contiguity following scaffolding indicate that divergent heterozygous overlaps can be a significant barrier to scaffolding, and that it is important to remove them as well as removing contained haplotigs. To our knowledge, scaffolders that use long-range information, such as Scaff10X with linked reads or SALSA with Hi-C data, do not handle heterozygous overlaps. We therefore recommend applying purge_dups directly after initial assembly, prior to scaffolding. Although HaploMerger2 can also link adjacent contigs using overlap information after purging, our tests suggest that it makes false joins, perhaps because it does not use read depth to distinguish haplotypic duplication from repeat duplication.

In conclusion, purge_dups can significantly improve genome assemblies by removing overlaps and haplotigs caused by sequence divergence in heterozygous regions. This both removes false duplications in primary draft assemblies while retaining completeness and sequence integrity, and can improve scaffolding. It runs autonomously without requiring user specification of cutoff thresholds, allowing it to be included in an automated assembly pipeline.

## Supplementary Material

btaa025_Supplementary_Data
