# Supplementary material for: Identifying and removing haplotypic duplication in primary genome assemblies
Source: Bioinformatics. 2020 Jan 23;36(9):2896–8. doi: 10.1093/bioinformatics/btaa025 (PMC7203741; doi:10.1093/bioinformatics/btaa025)
Supplement: btaa025_Supplementary_Data [file btaa025_supplementary_data.pdf]

Purge_dups supplementary note

Dengfeng Guan[1,2], Shane A. McCarthy[2], Jonathan Wood[3], Kerstin Howe[3], Yadong Wang[1], and Richard Durbin[2,3]

[1]Center for Bioinformatics, Harbin Institute of Technology, Harbin, 150001, China
[2]Department of Genetics, University of Cambridge, Cambridge, CB2 3EH, UK
[3]Wellcome Sanger Institute, Wellcome Genome Campus, Cambridge, CB10 1SA, UK

Contents

1 Supplementary Methods **1**
1.1 Read depth cutoffs calculation 1
1.2 Haplotypic duplication identification 1

2 Supplementary Data **2**
2.1 Datasets 2
2.2 Software tools 2
2.3 Purge_dups commands 3
2.4 Analysis parameters 3

3 Supplementary Tables and Figures **3**

1 Supplementary Methods

1.1 Read depth cutoffs calculation

Given a read depth histogram H, purge_dups calculates the read depth cutoffs with the following algorithm.

- Initially calculate differences $H' = H_{i+1} - H_i$ of H, then smooth these using their 10 nearest neighbours to approximate the local derivative.
- Next, use the smoothed derivatives to find the turning points.
- Next we consider two cases: (1) ≥ 2 maxima are found, or (2) single maximum.
- In case (1) we first merge local maxima and minima (within 3 bins). If following this merging there remain two maxima with a minimum in between then we take the minimum v as the threshold between haploid and diploid, with interval $(N, v]$ for haploid and $(v, 3v]$ for diploid, where N is the noise cutoff, user-configurable with default value 5. Otherwise we take the highest remaining maximum and drop into case (2).
- For case (2) we decide whether this single peak at p represents haploid or diploid depth by comparing it to the mean read depth. If the peak occurs at below mean read depth we consider it to be haploid and set the intervals as $(N, 1.5p]$ for haploid and $(1.5p, 4.5p]$ for diploid. If the peak is above the mean read depth then we take $(N, 0.75p]$ for haploid and $(0.75p, 2.25p]$ for diploid.

Purge_dups outputs the thresholds calculated, and also the particular decision process applied, including for the single peak case the peak and mean depths. When the mean is sufficiently far from the peak this works well, but when they are close, for example with very low heterozygosity samples, it can make a mistake. An assembly pipeline can therefore inspect this information and choose to flag marginal decisions for manual oversight. In case a user wants to change the default, there is a command line option to force treatment of the peak depth as either haploid or diploid according to the user's choice.

1.2 Haplotypic duplication identification

Given a matching set of all versus all self alignments from minimap2, and read depth cutoffs from the previous section, purge_dups uses the following steps to identify the haplotypic duplications in a draft primary assembly:

1. Contained haplotig identification: purge_dups uses essentially the same way as purge_haplotigs to detect the contained haplotigs. If more than 80% bases of a contig are above the high read depth cutoff or below the noise cutoff, it is binned into the potential junk bin. Otherwise if more than 80% bases are in the diploid depth interval it is labelled as a primary contig, otherwise it is considered further as a possible haplotig. Next for each possible haplotig, we consider its best alignment to another contig. If its alignment score is larger than s (default 70) and max match score larger than m (default 200), it is marked as a repeat and is placed in the haplotig bin; if the alignment score is larger than s and max match score not larger than m, it is marked as a haplotig and also placed in the haplotig bin. Otherwise it is left as a candidate primary contig.

2. Haplotypic overlap identification: after purging the junk and contained haplotigs, purge_dups chains the matches between remaining candidate primary contigs to find collinear matches with the following process (Supplementary Figure 5):

   i Given all matches between contig Q and contig T, purge_dups builds a direct acyclic graph (DAG) with the matches as vertices. Each vertex V_i in DAG is denoted as a tuple (s, e, h, t, d, m), where s and e are the start and end position on Q, h and t are the start and end position on T, d is the orientation and m is the number of matched bases.

   ii all vertices are ordered by their start positions on Q. For a pair of (V_i, V_j), suppose without loss of generality that V_i is a predecessor of $V_j$they are both aligned in the forward direction, and the overlap between V_i and V_j on Q is represented by $Q^o_{i,j}$, and on T is $T^o_{i,j}$. An edge exists between V_i and V_j if they meet the following conditions:

   $$\begin{cases} V^e_i < V^e_j, V^t_i < V^t_j, max(V^s_j - V^e_i, V^h_j - V^t_i) < g \\ Q^o_{i,j}/min(V^e_j - V^s_j, V^e_i - V^s_i) < 0.95 \\ T^o_{i,j}/min(V^t_i - V^h_i, V^t_j - V^h_j) < 0.95 \end{cases} \quad (1)$$

   Where g is the maximum allowed gap size. Once the DAG is built, purge_dups will find the local optimal path by dynamic programming using the following recurrence equation:

   $$S(j) = max\{S(i) + V^m_j\}, V_i \in predecessors(V_j) \quad (2)$$

   where S_j is the score of V_j.

   iii After merging all the colinear matches, purge_dups filters out all the nested matches and matches whose score is less than a threshold l (default: 10,000).

3. Calculate average read depth for the matching intervals in both the query and target, and only keep matches both of whose average read depths are below the diploid cutoff. Remove secondary and overlapping matches, defined as those for which the query region is contained within less than 85% of the matching region of another match from the same query, or no more than 85% of its sequence overlaps with another match. For remaining matches, move the sequence corresponding to the matching interval of the shorter contig into the haplotigs bin.

2 Supplementary Data

2.1 Datasets

Datasets used in the experiments are listed as follows:

- **At**: We used the same assemblies for *Arabidopsis thaliana* as used in the purge_haplotigs paper, available at `https://zenodo.org/record/1419699`. SRA accessions for Pacbio reads are SRR3405291-SRR3405298 and SRR3405300-SRR3405326, and for paired-end Illumina reads are SRR3703081, SRR3703082, SRR3703105.

- **Ac**: The draft *Anopheles coluzzii* primary assembly that we used is available at `https://drive.google.com/open?id=18osbKPOiUDWi65R5hpdbzNpGRgUtsJQy`, the accession ID of the raw Pacbio reads is SRR8291675, and the RefSeq Accession ID of the AgamP4 PEST assembly for *Anopheles gambiae* is GCA_000005575.2.

- **Vv**: We used the same assemblies for *Vinus vinera* as used in the purge_haplotigs paper, available at `https://zenodo.org/record/1419699`, the accession IDs of the raw Pacbio reads are SRR3321323 and SRR3321342-SRR3321414.

- **Mm**: The draft Pacbio primary assembly is available at `s3://genomeark/species/Myripristis_murdjan/fMyrMur1/assembly_cambridge/intermediates/falcon_unzip/fMyrMur1.PB.asm1.unzip.primary.fa.gz`. The reference genome accession ID is GCF_902150065.1. All the sequencing data are available in ENA with the sample id SAMEA4872133.

2.2 Software tools

The following software tools were used in the experiments:

Tools	Version	Usage	Source
purge_dups	V0.0.3	automatic haplotigs and overlaps purger	https://github.com/dfguan/purge_dups
purge_haplotigs	V1.0.4	semi-automatic haplotigs purger	https://bitbucket.org/mroachawri/purge_haplotigs
BUSCO	V3.1.0	genome assembly assessment tool	https://gitlab.com/ezlab/busco
KMC[1]	-	K-mer coverage plot tool	https://github.com/dfguan/KMC
cgplot	-	Dotplot script	https://github.com/dfguan/cgplot

1: modified from a k-mer counting tool **KMC** published in Kokot, M., Dlugosz, M., and Deorowicz, S. (2017). KMC 3: counting and manipulating k-mer statistics. *Bioinformatics*, 33(17):2759-2761.

2.3 Purge_dups commands

Given raw Pacbio reads alignment PAF files *pfs*, and a primary assembly *asm*, purge_dups uses the following commands to identify the haplotigs and overlaps:

`pbcstat $pfs` // generates files PB.base.cov for base-level read depth and PB.stat for read depth histogram

```
calcuts PB.stat > cutoffs 2> calcults.log
split_fa $asm > $asm.split.fa
minimap2 -xasm5 -DP $asm.split.fa $asm.split.fa > $asm.split.self.paf
purge_dups -2 -T cutoffs -c PB.base.cov $asm.split.self.paf > dups.bed 2> purge_dups.log
get_seqs dups.bed $asm > purged.fa 2> hap.fa
```

2.4 Analysis parameters

Read depth cutoffs for purge_haplotigs were set manually and are shown here together with the databases used for BUSCO analysis:

Assembly	purge_haplotigs read depth cutoffs (low/middle/high)	BUSCO database
At	25/97/250	embryophyta
Ac	10/150/700	diptera
Mm	5/42/125	actinopterygii
Vv	25/103/190	embryophyta

3 Supplementary Tables and Figures

Assembly	Heterozygosity (%)	Genome size (Mbp)
At	1.04	135
Ac	0.61	262
Mm	1.06	847
Vv	1.58	475

Supplementary Table 1: Heterozygosity and genome size estimates calculated by GenomeScope. For At and Mm these are from Illumina data and for Ac and Vv from PacBio CCS HiFi data.

	N50 (Mb)	NG50 (Mb)	NGA50 (Mb)	# Contig misassemblies	# Scaffold misassemblies
Mm-origS	4.88	6.96	3.46	5868	384
Mm-PDS	23.78	23.78	16.73	311	22
Mm-PHS	8.17	8.51	3.83	1102	178
Mm-HMS	26.79	26.79	7.38	1699	115
Mm-HMmS	34.53	34.53	7.86	1937	126

Supplementary Table 2: Scaffold N50, NG50, NGA50 measured by QUAST using the reference genome.

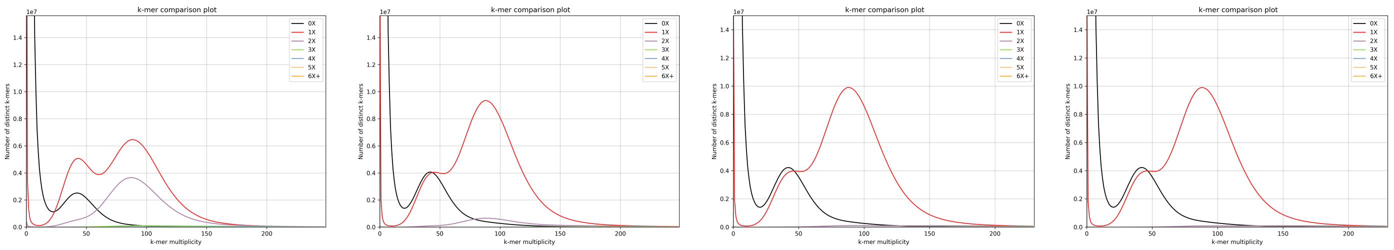

(a) (b) (c) (d)

Supplementary Figure 1: **K-mer coverage plots for draft and purged Mm assemblies (k=21).** The horizontal axis represents the copy number of k-mers in short reads from the same sample, the vertical axis shows the number of distinct k-mers, and the colored lines denote k-mers which occur the given number of times in the assembly. (**a**) The purple line shows 209.1 million 2-copy k-mers accumulating in the haploid and diploid areas, which correspond to duplicated haplotigs or overlaps in the primary assembly. (**b**) 39.3 million 2-copy k-mers remain after purging with purge_haplotigs. (**c**) 9.0 million 2-copy k-mers remain after processing with HaploMerger. (**d**) Only 7.6 million 2-copy k-mers remain after purging with purge_dups.

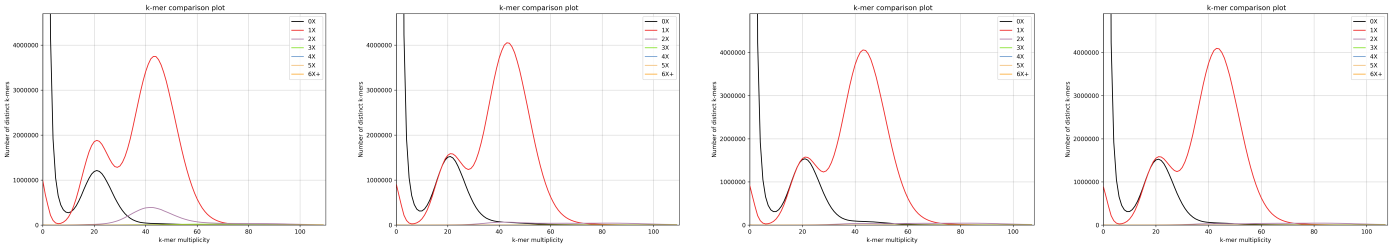

(a) (b) (c) (d)

Supplementary Figure 2: **K-mer coverage plots for the At primary and purged assemblies (k=21).** (**a**): 8.06 million 2-copy k-mers remain in the diploid area of the original assembly (purple line). (**b**): 1.56 million remain after purge_haplotigs. (**c**): 1.02 million remain after HaloMerger. (**d**): 0.94 million remain after purge_dups.
We can not make this plot for assembly Ac because we do not have Illumina data from the same sample.

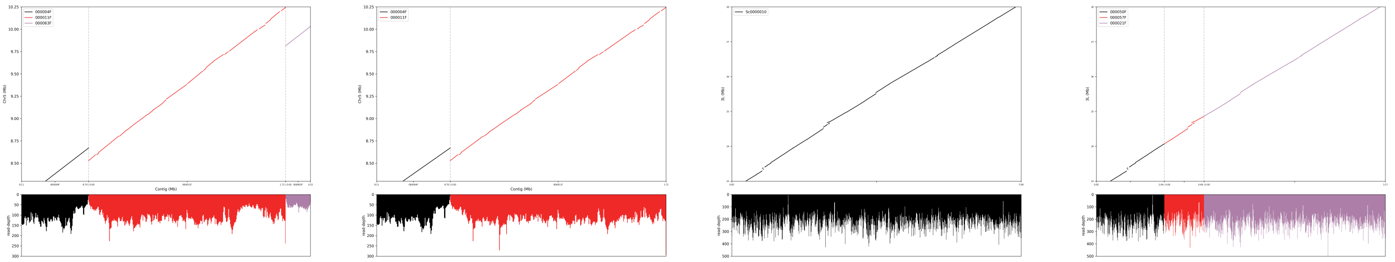

(a) (b) (c) (d)

Supplementary Figure 3: **Dotplots of draft and purged At assemblies mapped to the TAIR10 reference genome.** The horizontal axis represents the contigs in the assemblies, the upper vertical axis represents the reference chromosome, and the lower one shows the read depth for the contigs. (**a**) In the draft assembly, the right end of contig "000004F" and all of contigs "000011F" and "000063F" are aligned to part of chromosome 5. Contig "000063F" is contained in "000011F" and an overlap occurs at the ends of "000011F" and "000004F". The read depth at the haplotypic and overlapped region drops to almost half of the diploid read depth (150). (**b**) In the purge_haplotigs assembly, the haplotig is removed, and read depth at the haplotypic region goes back to diploid read depth. However the overlap remains. (**c**) In the HaploMerger assembly, both the haplotigs and overlaps are removed, and the read depth goes back to normal. (**d**) In the purge_dups assembly, both the haplotig and the overlap are removed and read depth goes back to normal across the whole range.

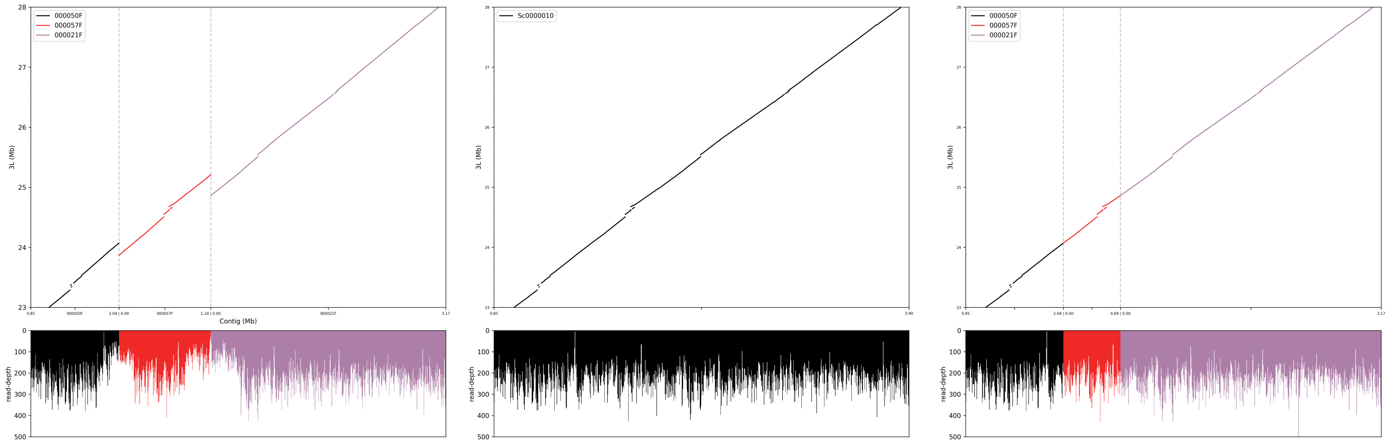

(a) (b) (c)

Supplementary Figure 4: **Dotplots on Ac draft primary and purge_dups assemblies** The horizontal axis represents the contigs in the assemblies, the upper vertical axis represents the reference chromosome, and the bottom one shows the read depth for the contigs. The draft and purged primary assemblies are mapped to AgamP4 PEST reference assembly. (**a**): Contig 50F, 21F and 57F are aligned to 23-28 Mb region of chromsome 3L on PEST genome. Two overlaps are found, the read depth of the corresponding regions also drops to half of the normal diploid coverage. (**b**): In the HaploMerger assembly, both overlaps are removed, and the read depth goes back to normal. (**c**): After purging with purge_dups, the overlaps are removed perfectly, and the read depth becomes even at the diploid level.

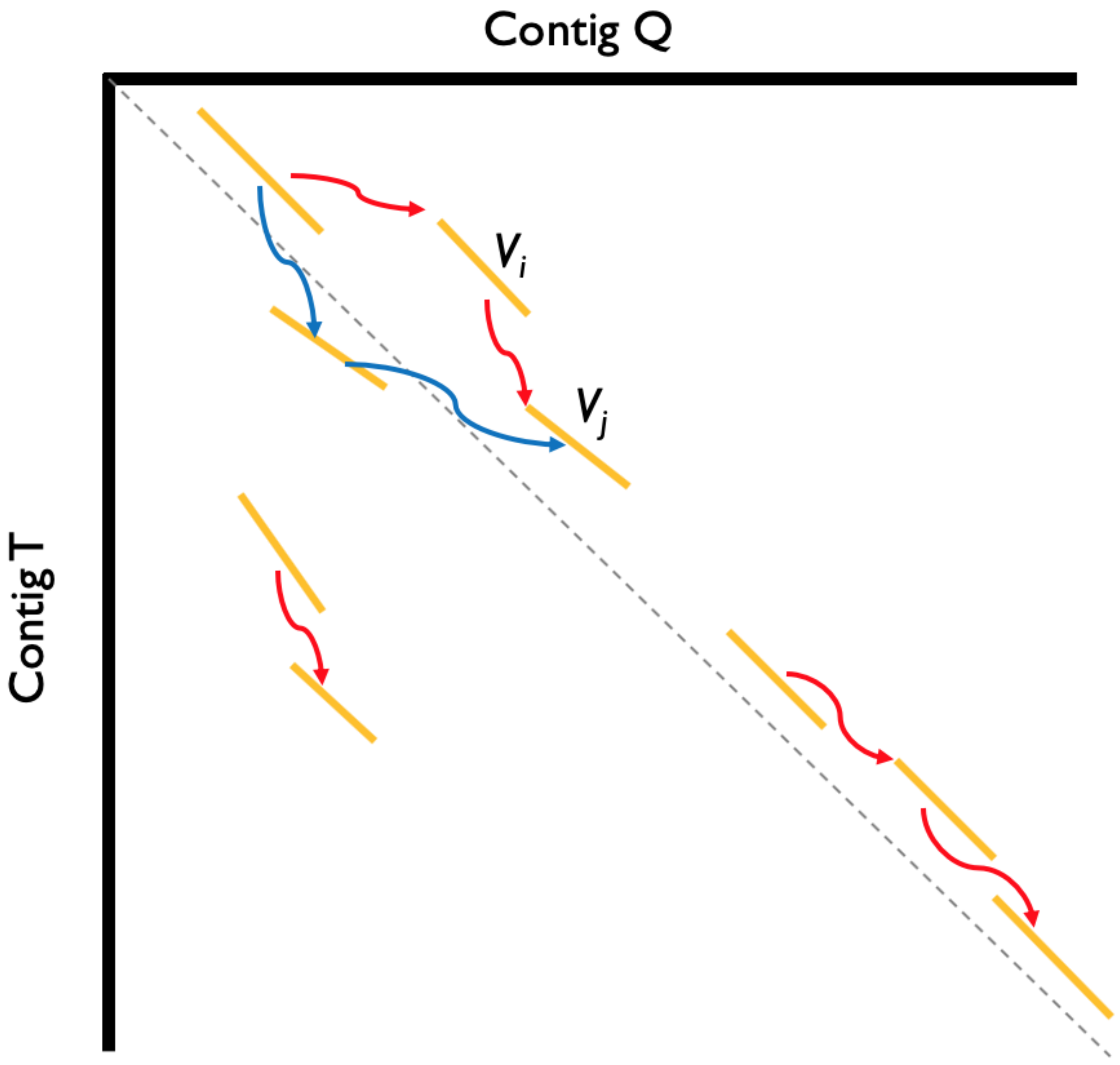

Supplementary Figure 5: **Illustration of chaining algorithm**. Vertices representing the matches are the lines in orange, edges are shown in red and blue. Red edges are used to form a collinear match. Three collinear matching groups are found in this example.
